# Focus-marking in a tonal language: Prosodic differences between Cantonese-speaking children with and without autism spectrum disorder

**DOI:** 10.1371/journal.pone.0306272

**Published:** 2024-07-19

**Authors:** Si Chen, Yixin Zhang, Fang Zhou, Angel Chan, Bei Li, Bin Li, Tempo Tang, Eunjin Chun, Zhuoming Chen

**Affiliations:** 1 Department of Chinese and Bilingual Studies, The Hong Kong Polytechnic University, Hong Kong SAR, China; 2 Department of Chinese and Bilingual Studies, Research Centre for Language, Cognition, and Neuroscience, The Hong Kong Polytechnic University, Hong Kong SAR, China; 3 Research Institute for Smart Ageing, The Hong Kong Polytechnic University, Hong Kong SAR, China; 4 The HK PolyU-PekingU Research Centre on Chinese Linguistics, The Hong Kong Polytechnic University, Hong Kong SAR, China; 5 Department of Linguistics and Translation, City University of Hong Kong, Hong Kong SAR, China; 6 Department of Rehabilitation Medicine, the First Affiliated Hospital of Jinan University, Guangzhou, China; National Taiwan Normal University, TAIWAN

## Abstract

Abnormal speech prosody has been widely reported in individuals with autism. Many studies on children and adults with autism spectrum disorder speaking a non-tonal language showed deficits in using prosodic cues to mark focus. However, focus marking by autistic children speaking a tonal language is rarely examined. Cantonese-speaking children may face additional difficulties because tonal languages require them to use prosodic cues to achieve multiple functions simultaneously such as lexical contrasting and focus marking. This study bridges this research gap by acoustically evaluating the use of Cantonese speech prosody to mark information structure by Cantonese-speaking children with and without autism spectrum disorder. We designed speech production tasks to elicit natural broad and narrow focus production among these children in sentences with different tone combinations. Acoustic correlates of prosodic focus marking like *f*_0_, duration and intensity of each syllable were analyzed to examine the effect of participant group, focus condition and lexical tones. Our results showed differences in focus marking patterns between Cantonese-speaking children with and without autism spectrum disorder. The autistic children not only showed insufficient on-focus expansion in terms of *f*_0_ range and duration when marking focus, but also produced less distinctive tone shapes in general. There was no evidence that the prosodic complexity (i.e. sentences with single tones or combinations of tones) significantly affected focus marking in these autistic children and their typically-developing (TD) peers.

## 1. Introduction

Autism Spectrum Disorder (henceforth ASD) is a heterogeneous neurodevelopmental disorder, characterized by pervasive abnormalities in social communication, repetitive behaviors and restricted interests [1]. Peculiar tones of voice and disturbances of prosody have been identified as the earliest characteristics of ASD. Children with ASD tend to show atypical patterns of speech prosody. While some earlier studies reported that autistic individuals may produce either monotonous or sing-songy prosody, more recent studies report that children with ASD tended to produce high-pitched and exaggerated prosody cross-linguistically (for English, see [2,3]; for German see [4,5]; For Cantonese, see [6]; for Hindi-English bilinguals, see [7]).

The research on prosody production among individuals with ASD is important because speech prosody is a key component in communication. It is also reported that prosodic impairments and social communication are strongly correlated [8] and impairments in speech prosody can negatively affect friends making and job seeking [9]. However, the existing research on prosody production in ASD, has been focusing on speakers of non-tonal languages, leaving the interaction between lexical tones and intonation in tone languages under-investigated (for a review see [10]). Tonal languages may offer a more challenging situation for individuals with ASD in using discourse functions such as focus marking because the acoustic cues such as fundamental frequency (*f*_0_) are used to achieve both lexical contrasts and focus marking. The present study aims to fill in this research gap by analyzing the acoustic features of focus-marking by Cantonese-speaking children with ASD in comparison with their typically developing (TD) peers. The results may improve our understanding of prosodic production deficits in the population with ASD and may have clinical implications.

### 1.1 Prosodic focus-marking in children with ASD

Speech prosody is the vocal modulation accompanying speech, which comprises variations in *f*_0_, duration, intensity and voice quality and serves a wide range of communication functions, such as signaling information structure and expressing the speakers’ emotions and attitudes [11]. A typical example of information structure categories is focus, which marks new information to the receiver(s) in a sentence, [12,13]. There are two main focus types: broad focus (i.e., focus falling on the entire utterance) and narrow focus (i.e., focus falling on a selective part of an utterance). Narrow focus can be further categorized into non-contrastive and contrastive narrow focus, with the latter providing an explicit contrast to alternatives [13]. Focus can be marked by morpho-syntactic and prosodic means. Acoustic correlates of focus on and beyond the components on focus have been reported. Despite language-specific differences, components on focus are often realized with longer duration, higher *f*_0_ values or larger *f*_0_ range, and/or increased intensity than the components carrying no focus (for English see [14,15], for German see [16], for Mandarin see [17], for Japanese see [18], and components following on-focus syllables are also realized with reduced *f*_0_ range and intensity (i.e., post-focus compression, PFC) in languages like English, Greek, Dutch, Korean, and Mandarin (for review, see [19]).

Children with ASD tend to show delayed, deviant development and deficits in speech prosody. Meta-analyses of acoustic studies on prosodic features of vocal productions suggest that speech prosody of the autistic population is characterized by significantly higher mean *f*_0_, larger *f*_0_ range, longer voice duration and greater *f*_0_ variability [10,20]. Differences between children with ASD and TD children in other acoustic parameters have also been reported in other studies. For instance, Patel et al. [21] reported slower speech rate for autistic individuals, while Bone et al. [22] reported a positive association between ASD severity and median *f*_0_ slope as well as atypical voice quality like jitter and shimmer. It is worth mentioning, however, there are also studies reporting no significant differences between the speech rate of individuals with and without ASD [23,24].

There is a paucity in research focusing on the production of prosodic prominence by autistic children. Several studies demonstrate that autistic children were able to produce stressed syllables with longer duration and sometimes larger intensity, but the contrastivity they demonstrated is often less evident or natural than their TD peers [25–30]. For instance, Paul et al. [25] and Grossman et al. [26] both found that English-speaking children with ASD have the knowledge to lengthen the stressed syllables just like their TD peers, but unlike their TD peers, the differences between stressed and unstressed syllables did not reach statistical significance.

In terms of prosodic focus marking, Diehl and Paul [3,31] also found that the differences between syllables carrying or not carrying focus in the autistic speech were less prominent than those in the TD speech. It is worth mentioning that in Diehl and Paul’s studies, children with ASD tended to over-lengthen the syllables carrying no focus, unlike those in Paul et al.’s study, who did not lengthen the stressed syllables enough. The differences may arise from the different tasks and stimuli used in these two studies. Paul et al. elicited speech via imitation using the Tennessee Test of Rhythm and Intonation Patterns (T-TRIP, [32]) which involved 25 prerecorded nonsense syllable /ma/ varying in rhythm and intonation. Diehl and Paul, however, used Profiling Elements of Prosodic Systems (PEPS-C), which assesses children’s abilities to discriminate and articulate the prosodic forms in four areas of communication where prosody plays a critical role, namely, interaction, affect, boundary and focus [33]. Studies using PEPS-C have generally reported a significantly worse performance of the autistic children than their TD peers in both perceptual and production tasks [31,34].

Meanwhile, there are also studies reporting comparable performance between the autistic and TD children. For instance, Nadig & Shaw [27] acoustically analyzed on- and post-focus syllables produced by English-speaking children with and without ASD and found that both groups produced significantly longer and louder on-focus syllables than post-focus ones, but neither of them used mean *f*_0_ in focus marking. The existing research has reported complex results in the use of *f*_0_ in focus marking by the autistic children. DePape et al. [35] found that it were the autistic children with moderate rather than high language skills that used *f*_0_ range to mark information structure, although children with moderate skills did not necessarily master the correct usage of *f*_0_ range, and their performance may be influenced by the intervention they previously received.

From the studies reviewed so far, it seems that the use of *f*_0_ cues by autistic children in focus marking, in particular, seems to be more problematic. This makes prosodic focus marking in tone-language speaking children with ASD an interesting topic, as they do not only need to make the components on focus acoustically more prominent but also to keep the shape of lexical tones so as to convey the core meanings of words, which remains to be explored.

### 1.2 Focus marking in Cantonese

Cantonese is a typical tone language that uses *f*_0_ to contrast meanings of words. There are six full tones (i.e. carried by open syllables) and three checked tones (i.e. carried by syllables ending with /p/, /t/ or /k/) in Cantonese. An example of all full tones on the [fu] syllables is given as follows: [fu] with Tone 1 (55/53) ‘to call’; Tone 2 (25) ‘bitter; Tone 3 (33) ‘rich’; Tone 4 (21) ‘to hold’; Tone 5 (23) ‘woman’; and Tone 6 (22) ‘rotten’ (the numbers in bracket are Chao Tone Numeral, which marks the lowest pitch point with 1 and the highest with 5) [36].

As mentioned earlier, prosodic marking of focus is usually manifested in acoustic cues such as *f*_0_, intensity and duration [15]. In addition to the adjustment of acoustic cues of on-focus words (e.g. higher *f*_0_ values, larger *f*_0_ range, longer duration and larger intensity), post-focus compression (i.e. reduced *f*_0_ range and intensity of words after the on-focus words [37]), has also been found in many languages. However, the acoustic correlates of focus marking in Cantonese remain controversial. Some studies report on-focus *f*_0_ expansion and post-focus *f*_0_ compression in Cantonese [38,39], but others suggest that prosodic prominence in Cantonese is primarily signaled by on-focus lengthening [40,41]. For instance, Mann [39] examined the *f*_0_ changes of Cantonese monosyllabic words in broad and narrow focus conditions and found an expansion of *f*_0_ range for narrow focus, and yet the expansion may be affected by tone-focus interaction. However, using six sentences with the same tones on each syllable (from all Tone 1, all Tone 2 up to all Tone 6), Wu and Xu [42] found an increment of *f*_0_ excursion size in the dynamic tones but no increment in the static tones, and they reported no post-focus compression for Cantonese. In a more recent study, Fung and Mok [40] found no significant on-focus *f*_0_ changes, arguing that corrective focus in Cantonese is marked solely by durational expansion. The perceptual research, though relatively rare, is more in line with Fung and Mok’s studies, suggesting that Cantonese speakers rely on longer duration in prominence perception [43].

The mixed results regarding on-focus *f*_0_ changes in typical population allows us to come up with a concrete hypothesis as follows: it is possible that the Cantonese-speaking children with ASD encounter more difficulties when producing focus than their non-tone language speaking peers as they need to produce lexical tones accurately while making proper exaggeration and/or compression of the *f*_0_ height and contours. On-focus lengthening may also be difficult for the autistic children since studies reviewed in Section 1.1 also showed abnormal use of duration in stress marking among the population with ASD.

### 1.3 The current study

The literature reviewed so far indicated that children with ASD speaking tonal languages may face greater difficulties as the same prosodic cue *f*_0_ need to encode both lexical and intonational functions, but the focus marking and the effects of tones on it have not been investigated. The current study is the first study that attempts to fill in this gap by investigating prosodic focus marking by Cantonese-speaking children with ASD. Specifically, this study aims to answer the following questions: 1) What prosodic cues are employed in focus marking by Cantonese-speaking children with and without ASD? Do the two groups differ in using cues to mark focus? 2) Is the focus marking by autistic and non-autistic children affected by tones? Is focus marking by these two groups of children affected by tones differently and if so, how?

The results may further our understanding about prosody-related deficits by providing new evidence from a tonal language. It is also worth mentioning that we used a different paradigm from the widely used PEPS-C, that is, we elicited spontaneous focus production from children using specifically designed games to ensure the naturalness of the speech production. In this way, focus marking in speech production is investigated separately and not influenced by a preceding speech perception task like in the PEPS-C paradigm.

## 2. Methodology

### 2.1 Participants

Twenty-three native Cantonese-speaking children with ASD (19 males and 4 females) and twenty-three Cantonese TD children (19 males and 4 females) participated in the experiment. All of the ASD participants in the experiment were formally diagnosed with ASD by professionals in established institutions based on ADOS-2 and other assessments. No participants were diagnosed of or suspected to have any other disorders. No TD participants had any speech or language disorders or suspected to have any disorders. Participants were invited to the speech laboratory at the Hong Kong Polytechnic University accompanied by parents. All child participants and parents were well-informed and agreed to participate in the experiment. Written consent was obtained from parents of child participants and verbal consent was obtained from child participants. The parents signed the consent forms of a protocol approved by the Human Subjects Ethics Sub-committee at the Hong Kong Polytechnic University on behalf of the child participants, and they also filled in questionnaires on the demographic and clinic conditions (if applied) of the children. All protocols were carried out in accordance with relevant guidelines and regulations. All participants were compensated for participating in the experiment.

ASD and TD participants with and without ASD were matched in age, gender, linguistic background and musical training background. The demographic information of the participants is summarized in Table 1. All participants spoke Cantonese as their first and dominant language at home and school.

**Table 1:** Demographic information and test scores of the participants.

Group	ASD	TD
Age	8.08 ± 1.49	7.82 ± 1.16
Age Range	From 6 to 10.8	From 6 to 10.67
English Learning (Age)	2.92 ± 1.03	3.87 ± 0.96
Mandarin Learning (Age)	4.06 ± 1.75	3.56 ± 0.99
Musical Training (Age)	6.27 ± 1.34	5.32 ± 1.01
Musical Training (Duration)	1.94 ± 1.26	2.54 ± 1.75
Raven’s Progressive Matrices (IQ score)	109.87 ± 16.00	113.61 ± 13.82
HKCOLAS score (Narration)	78.96 ± 23.12	86.57 ± 26.34
HKCOLAS score (Expressive Naming)	57.83 ± 18.97	58.26 ± 23.89

### 2.2 Tests

All participants were formally tested using the verbal language tests (expressive naming and narration) in Hong Kong Cantonese Oral Language Assessment Scale (HKCOLAS) [44] and the non-verbal analytical intelligence with the Raven’s Progressive Matrices (IQ) [45]. The standard scores and age equivalent were obtained. HKCOLAS is a standardized speech and language assessment tool for Cantonese-speaking children. Two subtests (Narrative Test and Expressive Nominal Vocabulary Test) from HKCOLAS were used to assess the participants’ language ability in the current study. Raven’s Progressive Matrices test is a non-verbal intelligence test to assess abstract reasoning. There are sixty multiple choice questions on pattern matching. All questions were grouped into five sets, and within each set the questions were presented in an order where the difficulty of each set increased.

Tests results were also summarized in Table 1. We conducted t-tests and found no significant differences between the participants with and without ASD in Raven’s Progressive Matrices (IQ score) [*t*(44) = -0.85 *p* = 0.41], HKCOLAS score (Narration) [*t*(44) = -1.041, *p* = 0.30] and HKCOLAS score (Expressive Naming) [*t*(44) = -0.068, *p* = 0.95].

### 2.3 Stimuli

In total, 15 target sentences were used in the experiment. Each sentence contains five monosyllabic words. They all depict an action and have a subject, a verb and an object. The prosodic complexity of stimuli is controlled by using two types of sentences: sentences with all words bearing the same tone (one from the six tones: Tone 1, Tone 2, Tone 3, Tone 4, Tone 5 and Tone 6), and sentences with a mixture of tones in which subjects carried one tone while the verbs and objects carried a different tone. All the stimuli can be found in S1 File.

Fifteen corresponding pictures depicting the content of the target sentences were used to elicit natural answers from participants. Target sentences were grouped into five blocks and each block contains three target sentences. All the stimuli were presented randomly to each participant and the order of blocks was also randomized. For each sentence, a series of questions were designed to elicit the desired types of focus (i.e. broad, narrow and contrastive focus) in initial (subject), middle (verb), or final (object) positions.

The experimental session was made up of five blocks and each block contained 42 randomized trials [3 out of 15 target sentences * (1 broad focus + 1 non-contrastive narrow focus * 3 positions + 1 contrastive narrow focus * 3 positions) * 2 repetitions]. In total, 210 target sentences (42 trials * 5 blocks) were collected for each participant. The experiment was programmed in E-prime 2.0 [46].

### 2.4 Procedure

Experiments were conducted in a sound-proof booth at the speech lab of the Hong Kong Polytechnic University. Audio Technica ATone 2035 condenser microphone and Steinberg UR22mkII USB Audio Interface were used to record participants’ speech production with the sample rate of 44100 Hz in Audacity [47].

Every block consisted of a practice session and a test session. During the practice session, the participants were instructed to familiarize themselves with the pictures of people and animals performing different actions so that they could consistently label people, animals, and the actions depicted in order to successfully play the game. Then they repeated each sentence recorded by a native Cantonese-speaking female speech therapist aged 23 in the same lab. The practice helped to reduce production errors in the later experiment. We reduced the memory load by using three stimulus sentences in each block so that children were able to remember the sentences describing the pictures with no errors. The order of blocks was counterbalanced across participants within each group and all the trials in each session were presented randomly by the software E-prime 2.0.

**Fig 1:**
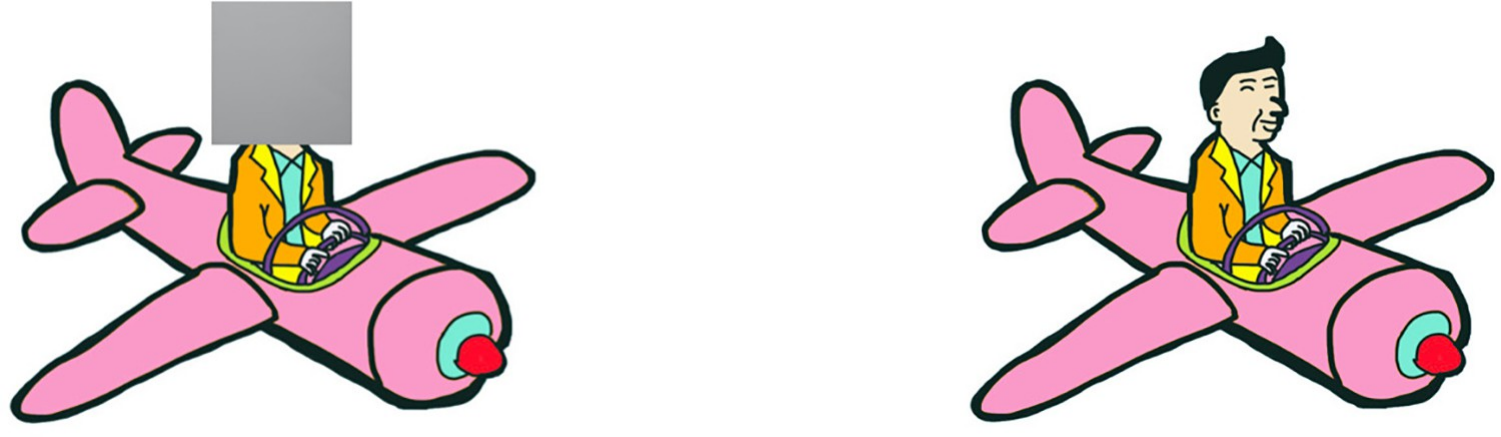
Illustration of the game “under the shape”. The sentence describes here is 張生揸飛機 "Mr. Cheung is operating an airplane", where all the words have Cantonese Tone 1.

During the experimental session, we followed the design of the game "under the shape" [48]. In each trial, the participants were presented with a sequence of pictures on the computer screen, and they needed to answer the question asked by the experimenter according to the picture (Fig 1).

For each sentence, a series of questions were designed to elicit each desired types of focus, namely, broad focus, non-contrastive narrow focus, and contrastive narrow focus. The positions of focus are initial, middle, or final positions. One picture covered by a grey shape was presented to participants in each trial. The experimenter will proceed to ask a question about the presented pictures. For example, in Fig 1, the participants were presented with the picture with a grey shape covering the person flying an airplane, and the experimenter asked in Cantonese, "Who is operating an airplane?" Then, the experimenter pressed a button and the grey shape on the picture was removed. The participant was then expected to answer the experimenter’s question by saying "Mr. Cheung is operating an airplane" with a focus on the subject. If a participant made a mistake in answering the question, namely, did not use the five-syllable answer required, the experimenter would ask the question again rather than simply ask for a correction so as to elicit a natural response. The maximum number of attempts was three, and none of the participants failed to correct themselves in this experiment.

### 2.5 Data extraction and analyses

In total, 9660 target sentences (15 sentences * 7 conditions * 2 repetitions * 23 participants * 2 groups) were acoustically analyzed for *f*_0_, duration and intensity. The five syllables of each sentences were manually segmented using *Praat* [49], following the procedure of segmentation written by Jangjamras [50]. Obstruents were not included into the segmentation and we focused only on the sonorant parts of the syllables. The data were extracted using ProsodyPro [51], and abnormal data were mannually checked by the first and second authors. In total, 5285 syllables were removed from the 48300 syllables due to creakiness and other abnormality. None of the participants had data loss larger than 20 percent.

The *f*_0_ range (i.e. the difference between maximum and minimum *f*_0_), the mean *f*_0_, the duration and mean intensity of the sonorant part were calculated for each syllable in each sentence. These four acoustic parameters were treated as the dependent (i.e., outcome) variables as they are widely used in prosodic marking cross-linguistically. The two *f*_0_ parameters can also index children’s performance of tone realization.

For independent (i.e., explanatory) variables, we were interested in the influence of *Participant Group* (i.e. ASD vs. TD), *Focus Condition* of the syllables, *Tone Shape*, *Prosodic Complexity* of the sentence and their interaction. *Focus Condition* was defined as the relative position to focus of a syllable, that is, 1) carrying broad focus (i.e. On-broad-focus), 2) preceding a syllable carrying contrastive or non-contrastive narrow focus (i.e. Pre-narrow-focus), 3) carrying narrow focus (i.e. On-narrow-focus), and 4) following a syllable carrying contrastive or non-contrastive narrow focus (i.e. Post-narrow-focus). Here contrastive and non-contrastive focus were not further separated in the analyses since these two types did not show significant differences. *Tone Shape* refers to the shape of tones carried by each syllable, which was grouped into 1) Non-low Level (Tone 1 and 3), 2) Rising (Tone 2 and 5) and 3) Low (Tone 4 and 6) tones. *Prosodic Complexity* was defined based on the tonal combination of the answers, which was grouped into 1) Single-tone (i.e. the five syllables in an answer carries the same tone) and 2) Mixed-tone (i.e. the two subject syllables carries a different tone from the verb and object syllables in an answer).

Linear mixed effects (LME) models were fitted to evaluate the fixed effects and their interactions on the four outcome variables using *lmer4* package (Bates et al., 2015) in R [52]. The optimal fixed structure of each model was selected by stepwise comparisons from the simplest structure to the most complex, and Likelihood Ratio (LR) tests were used to determine whether including factors from the analysis led to a better fit. Tukey post-hoc tests were used for post-hoc comparisons of the interactions of interests using *emmeans* [53]. Since mean *f*_0_ was not significantly affected by *Participant Group* nor was its interaction with other fixed effects significant, the results were not reported below.

## 3. Results

### 3.1 *F*_0_ range

Evaluation of the LME model showed that the inclusion of *Focus condition* [*χ*^2^ (3) = 41963, p < .0001], *Tone Shape* [*χ*^2^ (2) = 54088, *p* < .0001] and *the three-way interaction between Participant Group*, *Focus Condition* and *Tone Shape* [*χ*^2^ (6) = 28918, *p* < .0005] significantly contributed to the model (Table 2).

**Table 2:** LME model on *f*_0_ range (Significant results were highlighted with bold and italic fonts).

***F***_**0**_ **Range** ~ Participant Group+Focus Condition+Tone Shape+Participant Group:Focus Condition+Participant Group:Tone Shape+Focus Condition:Tone Shape+Participant Group:Focus Condition:Tone Shape+(1|Word)+(1|Subject)					
**Predictors**	**Estimates**	***CI***	**Statistics**	**df**	***p***
**(Intercept)**	29.61	23.13–36.10	8.95	46088	*<0*.*001*
**Participant Group (Baseline: ASD)**					
**TD**	0.66	-7.11–8.43	0.17		0.868
**Focus Condition (Baseline: On-broad-focus)**					
**Pre-narrow-focus**	-1.45	-3.57–0.66	-1.35	46088	0.177
**On*-*narrow*-*focus**	-1.76	-3.81–0.29	-1.68	46088	0.092
***Post-narrow*-*focus***	***-3*.*62***	***-5*.*73 –-1*.*51***	***-3*.*36***	***46088***	***0*.*001***
**Tone Shape (Baseline: Level)**					
**Rising**	***16*.*77***	***10*.*84–22*.*70***	***5*.*54***	***46088***	***<0*.*001***
***Low***	*6*.*54*	*-0*.*10–13*.*18*	*1*.*93*	*46088*	*0*.*053*
**Participant Group: Focus Condition (Baseline: TD: On-broad-focus)**					
**TD: Pre*-*narrow*-*focus**	1.65	-1.29–4.58	1.1	46088	0.272
***TD*: *On-narrow*-*focus***	***3*.*07***	***0*.*14–6*.*01***	***2*.*05***	***46088***	***0*.*04***
***TD*: *Post-narrow*-*focus***	***3*.*69***	***0*.*76–6*.*63***	***2*.*46***	***46088***	***0*.*014***
**Participant Group: Tone Shape (Baseline: TD: Level)**					
**TD: Rising**	1.51	-2.25–5.28	0.79	0.431	46088
***TD*: *Low***	***18*.*29***	***13*.*98–22*.*60***	***8*.*31***	***<0*.*001***	***46088***
**Focus Condition: Tone Shape (Baseline: On-broad-focus: Level)**					
**Pre*-*narrow*-*focus: Rising**	-2.86	-6.12–0.40	-1.72	46088	0.086
**On*-*narrow*-*focus: Rising**	-2.04	-5.25–1.18	-1.24	46088	0.215
***Post-narrow*-*focus*: *Rising***	***-5*.*35***	***-8*.*76 –-1*.*94***	***-3*.*07***	***46088***	***0*.*002***
***Pre-narrow*-*focus*: *Low***	***8*.*81***	***4*.*80–12*.*83***	***4*.*3***	***46088***	***<0*.*001***
***On-narrow*-*focus*: *Low***	***7*.*76***	***4*.*05–11*.*47***	***4*.*1***	***46088***	***<0*.*001***
***Post-narrow*-*focus*: *Low***	***7*.*12***	***3*.*43–10*.*82***	***3*.*78***	***46088***	***<0*.*001***
**Group: Focus Condition: Tone Shape (Baseline: TD: On-broad-focus: Level)**					
**TD: Pre*-*narrow*-*focus: Rising**	-0.42	-4.97–4.12	-0.18	46088	0.855
**TD: On*-*narrow*-*focus: Rising**	0.35	-4.27–4.96	0.15	46088	0.883
**TD: Post*-*narrow*-*focus: Rising**	2.13	-2.57–6.84	0.89	46088	0.373
***TD*: *Pre-narrow*-*focus*: *Low***	***-9*.*58***	***-15*.*09 –-4*.*06***	***-3*.*4***	***46088***	***0*.*001***
***TD*: *On-narrow*-*focus*: *Low***	***-9*.*92***	***-15*.*22 –-4*.*62***	***-3*.*67***	***46088***	***<0*.*001***
***TD*: *Post-narrow*-*focus*: *Low***	***-10*.*68***	***-15*.*85 –-5*.*51***	***-4*.*05***	***46088***	***<0*.*001***

**Fig 2:**
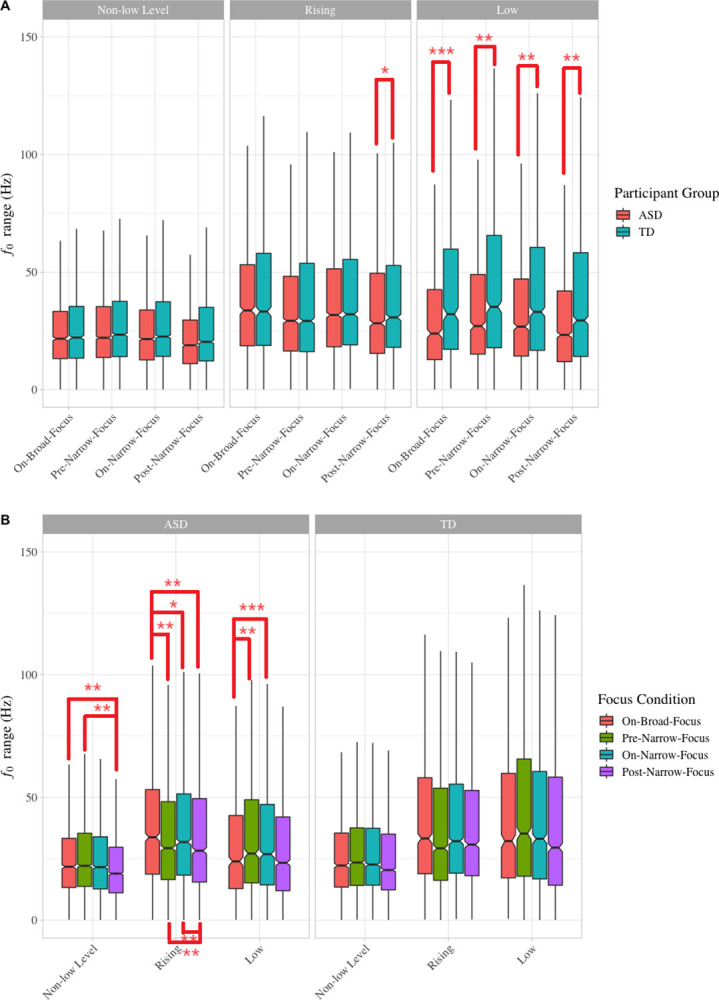
Boxplots of *F*_0_ range. Note. Statistically significant differences between specific comparisons are indicated by asterisk: * indicates p < .05, ** indicates p < .01, and *** indicates p < .001.

Post-hoc comparisons showed significant between-group differences mainly when Tone Shape was low tone. The *f*_0_ range of low tones produced by the children with ASD was significantly smaller than that produced by TD children in the two on-focus conditions (On-broad-focus, *p* < 0.0001; On-narrow-focus, *p* < 0.01) as well as in the two no-focus conditions (*p*s < 0.005). On non-low level or rising tones, the children with ASD also produced smaller *f*_0_ range than their TD counterparts, but the difference was only significant in post-narrow-focus syllables carrying rising tones (*p* < 0.05) (Fig 2A).

Post-hoc comparisons also showed within-group differences between focus conditions, indicating different focus-marking strategies used by the two groups (Fig 2B). In general, when examined by lexical tones, only in the ASD group were the differences between focus conditions statistically significant. When carrying non-low level tones, the *f*_0_ range of post-narrow-focus syllables produced by the autistic children were smaller than syllables in other focus conditions and the differences between post-narrow-focus and syllables on narrow and broad focus were significant (*p*s < 0.005). By contrast, no significant differences were found between focus conditions in the TD group. When carrying rising tones, the *f*_0_ range of post-narrow-focus syllables produced by the autistic children were the smallest, followed by that of pre-narrow-focus, on-narrow-focus and on-broad-focus syllables, and all these differences were significant except for those between pre- and on-narrow-focus syllables (On-broad-focus vs. On-narrow-focus: *p* < 0.05; Others: *p*s < 0.005). In the TD group, however, the smallest *f*_0_ range was found in pre-narrow-focus syllables, while no differences were found between syllables on broad focus and syllables on narrow focus; only on-narrow-focus syllables were marginally larger than pre-narrow-focus syllables (*p* = 0.052). When carrying low tones, in the ASD group, on-broad-focus syllables had the smallest *f*_0_ range, which was significantly smaller than that of the pre-narrow focus syllables (*p* < 0.005) and on-narrow focus syllables (*p* < 0.001). In the TD group, it was the post-narrow-focus syllables that had the smallest *f*_0_ range and the on-broad-focus syllables that had the largest, but no statistical significance was found.

### 3.2 Duration

Evaluation of the LME model showed that the inclusion of *Focus condition* [*χ*^2^ (3) = 718474, p < .0001], *Prosodic Complexity* [*χ*^2^ (1) = 357523, p < .0001] and *the three-way interaction between Participant Group*, *Focus condition* and *Tone Shape* [*χ*^2^ (6) = 109221, *p* < .05] significantly contributed to the model (Table 3).

**Table 3:** LME model on duration (Significant results were highlighted with bold and italic fonts).

**Duration** ~ Participant Group+Focus Condition+Tone Shape+Participant Group:Focus Condition+Participant Group:Tone Shape+Focus Condition:Tone Shape+Participant Group:Focus Condition:Tone Shape+(1|Word)+(1|Subject)					
**Predictors**	**Estimates**	***CI***	**Statistics**	**df**	***p***
***(Intercept)***	***209*.*09***	***183*.*02–235*.*16***	***15*.*72***	***45448***	***<0*.*001***
***Participant Group (Baseline*: *ASD)***					
***TD***	0.3	-17.93–18.53	0.03	45448	0.974
***Focus Condition (Baseline*: *On-broad-focus)***					
***Pre-narrow-focus***	-2.25	-7.42–2.92	-0.85	45448	0.394
***On-narrow-focus***	-0.02	-5.04–5.00	-0.01	45448	0.994
***Post-narrow-focus***	***-8*.*54***	***-13*.*71 –-3*.*37***	***-3*.*24***	***45448***	***0*.*001***
***Tone Shape (Baseline*: *Level)***					
***Rising***	***42*.*91***	***8*.*18–77*.*64***	***2*.*42***	***45448***	***0*.*015***
***Low***	33.8	-4.88–72.48	1.71	0.087	45448
***Participant Group*: *Focus Condition (Baseline*: *TD*: *On-broad-focus)***					
***TD*: *Pre-narrow-focus***	-4.97	-12.26–2.32	-1.34	45448	0.181
***TD*: *On-narrow-focus***	-4.45	-11.71–2.81	-1.2	45448	0.229
***TD*: *Post-narrow-focus***	***-7*.*59***	***-14*.*83 –-0*.*35***	***-2*.*06***	***45448***	***0*.*04***
***Participant Group*: *Tone Shape (Baseline*: *TD*: *Level)***					
***TD*: *Rising***	-6.05	-15.31–3.20	-1.28	45448	0.2
***TD*: *Low***	-0.17	-10.85–10.51	-0.03	45448	0.975
***Focus Condition*: *Tone Shape (Baseline*: *On-broad-focus*: *Level)***					
***Pre-narrow-focus*: *Rising***	***-8*.*67***	***-16*.*66 –-0*.*69***	***-2*.*13***	***45448***	***0*.*033***
***On-narrow-focus*: *Rising***	***-8*.*72***	***-16*.*59 –-0*.*85***	***-2*.*17***	***45448***	***0*.*03***
***Post-narrow-focus*: *Rising***	-6.33	-14.68–2.02	-1.49	45448	0.137
***Pre-narrow-focus*: *Low***	9.45	-0.39–19.29	1.88	45448	0.06
***On-narrow-focus*: *Low***	***14*.*95***	***5*.*88–24*.*03***	***3*.*23***	***45448***	***0*.*001***
***Post-narrow-focus*: *Low***	***15*.*61***	***6*.*56–24*.*67***	***3*.*38***	***45448***	***0*.*001***
***Group*: *Focus Condition*: *Tone Shape (Baseline*: *TD*: *On-broad-focus*: *Level)***					
***TD*: *Pre-focus*: *Rising***	***12*.*74***	***1*.*55–23*.*92***	***2*.*23***	***45448***	***0*.*026***
***TD*: *On-narrow-focus*: *Rising***	8.93	-2.40–20.26	1.55	45448	0.122
***TD*: *Post-narrow-focus*: *Rising***	2.91	-8.62–14.45	0.49	45448	0.621
***TD*: *Pre-narrow-focus*: *Low***	-9.56	-23.18–4.06	-1.38	45448	0.169
***TD*: *On-narrow-focus*: *Low***	***-13*.*86***	***-26*.*97 –-0*.*76***	***-2*.*07***	***45448***	***0*.*038***
***TD*: *Post-narrow-focus*: *Low***	***-14*.*08***	***-26*.*87 –-1*.*28***	***-2*.*16***	***45448***	***0*.*031***

**Fig 3:**
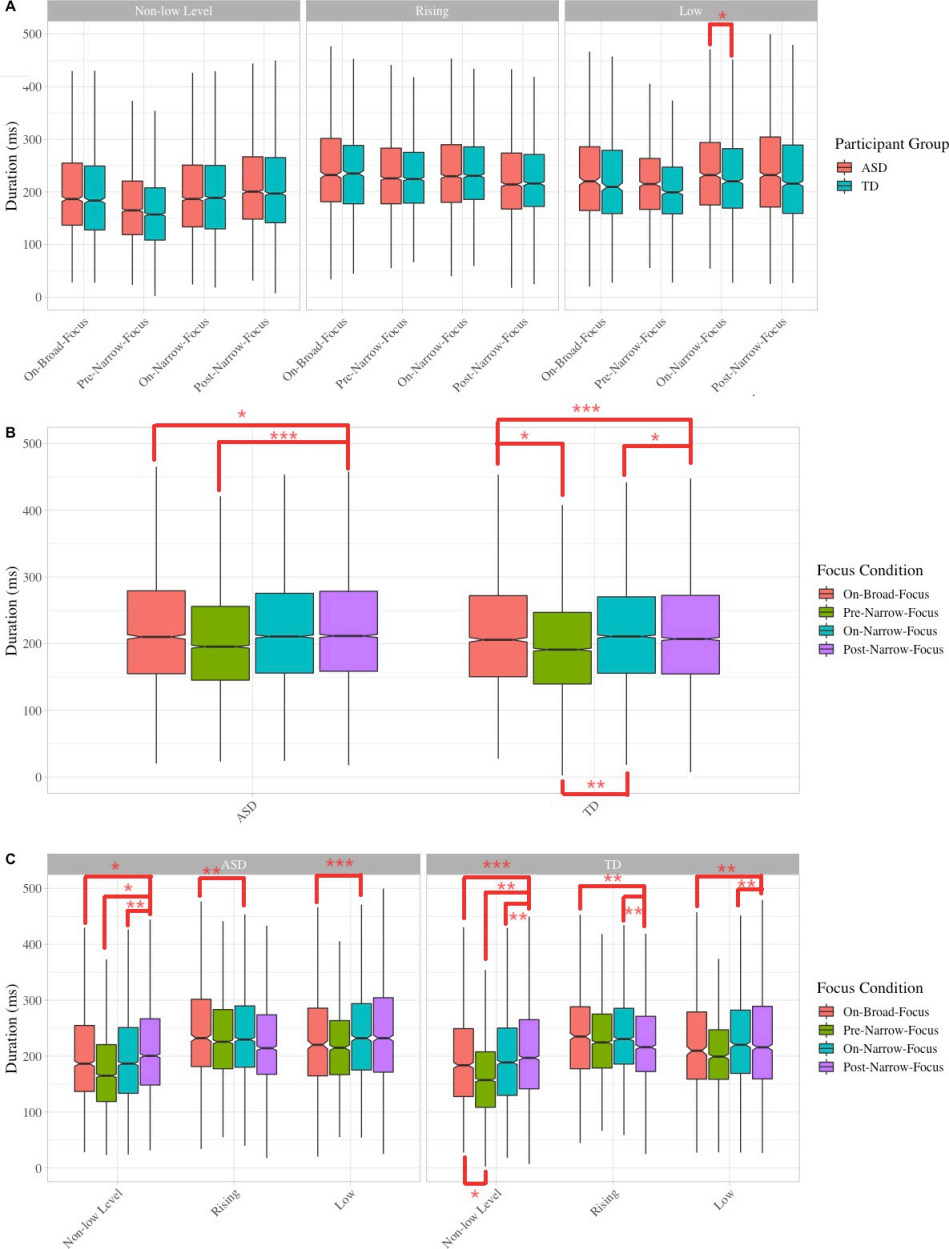
Boxplots of duration. Note. Statistically significant differences between specific comparisons are indicated by asterisk: * indicates p < .05, ** indicates p < .01, and *** indicates p < .001.

Like *f*_0_ range, significant differences between the ASD and TD groups were also found when on low tones, namely, the children with ASD produced significantly longer post-narrow-focus (*p* < 0.05) and marginally longer on-narrow-focus syllables than TD peers (*p* = 0.053) (Fig 3A).

With regard to within-group focus marking patterns (Fig 3B), the autistic children produced the longest duration in the post-narrow-focus syllables and shortest in the pre-narrow-focus syllables (*p* < 0.0001), while syllables on broad and narrow focus had similar mean duration, both significantly or marginally significantly shorter than post-narrow-focus syllables (*p* < 0.05; *p* = 0.052). In the TD group, by contrast, duration of syllables on broad focus was the longest, significantly longer than pre-narrow-focus (*p* < 0.05) and post-narrow-focus syllables (*p* < 0.0001); post-narrow-focus syllables were also significantly longer than on-narrow-focus syllables but shorter than on-narrow-focus syllables (*p*s < 0.005). The longer post-narrow-focus syllables found in both groups may be due to final lengthening, as many post-narrow-focus syllables were the last two syllables of the five-syllable stimulus sentences.

*Tone Shape* also influences the uses of duration in focus marking in the ASD and TD groups (Fig 3C). With regard to syllables carrying non-low level tones, in both groups, post-narrow-focus syllables were significantly longer than pre-narrow-focus (ASD, *p* < 0.05; TD, *p* < 0.005), on-narrow-focus (ASD, *p*s < 0.001) and on-broad-focus syllables (ASD, *p* < 0.01; TD, *p* < 0.001), and in the TD group, on-broad-focus syllables were also significantly longer than pre-narrow-focus (*p* < 0.05). With regard to syllables carrying rising tones, syllables on broad focus were significantly longer than those on narrow focus in the ASD group, whereas in the TD group, post-narrow-focus syllables were significantly shorter than syllables on broad and narrow focus (*p*s < 0.005). With regard to syllables carrying low tones, in the ASD group, syllables on broad focus were significantly shorter than those on narrow focus (*p* < 0.001) but in the TD group, duration of post-narrow-focus syllables were significantly shorter than on-narrow-focus syllables and on-broad-focus ones (*p*s < 0.005).

### 3.3 Intensity

Evaluation of the LME models showed that the inclusion of *Focus condition* [*χ*^2^ (3) = 1511.76, p < .0001], *Tone Shape* [*χ*^2^ (2) = 429.16, p < .0001], *Prosodic Complexity* [*χ*^2^ (1) = 2684.22, p < .0001] and *the three-way interaction between Participant Group*, *Focus condition* and *Tone Shape* [*χ*^2^ (12) = 764.80, *p* < .05] significantly contributed to the model (Table 4).

**Table 4:** LME model on intensity (Significant results were highlighted with bold and italic fonts).

**Intensity** ~ Participant Group+Focus Condition+Tone Shape+Participant Group:Focus Condition+Participant Group:Tone Shape+Focus Condition:Tone Shape+Participant Group:Focus Condition:Tone Shape+(1|Word)+(1|Subject)					
**Predictors**	**Estimates**	***CI***	**Statistics**	**df**	***p***
**(Intercept)**	61.44	58.75–64.13	44.73	46083	<0.001
**Participant Group (Baseline: ASD)**					
**TD**	1.49	-2.18–5.17	0.8	46083	0.425
**Focus Condition (Baseline: On-broad-focus)**					
**Pre-narrow-focus**	-0.2	-0.49–0.09	-1.34	46083	0.179
**On-narrow-focus**	0.06	-0.22–0.34	0.44	46083	0.658
**Post-narrow-focus**	-0.44	-0.73 –-0.15	-2.97	46083	0.003
**Tone Shape (Baseline: Level)**					
**Rising**	-0.56	-1.80–0.68	-0.89	46083	0.375
***Low***	***-3*.*51***	***-4*.*90 –-2*.*12***	***-4*.*94***	***46083***	***<0*.*001***
**Prosodic Complexity (Baseline: Single-tone)**					
**Mixed-tone**	3.35	2.85–3.84	13.17	46083	<0.001
**Participant Group: Focus Condition (Baseline: ASD: On-broad-focus)**					
**TD: Pre-narrow-focus**	-0.23	-0.61–0.14	-1.22	46083	0.223
**TD: On-narrow-focus**	-0.02	-0.39–0.36	-0.08	46083	0.933
**TD: Pos-narrow-focus**	-0.02	-0.39–0.36	-0.09	46083	0.926
**Participant Group: Tone Shape (Baseline: ASD: Level)**					
**TD: Rising**	-0.2	-0.68–0.28	-0.83	46083	0.408
**TD: Low**	0.36	-0.19–0.91	1.28	46083	0.2
**Participant Group: Prosodic Complexity (Baseline: ASD:Single-tone)**					
***TD*: *Mixed-tone***	***-1***	***-1*.*21 –-0*.*80***	***-9*.*52***	***46083***	***<0*.*001***
**Focus Condition: Prosodic Complexity (Baseline: On-broad-focus: Single-tone)**					
**Pre-narrow-focus: Mixed-tone**	-0.26	-0.62–0.09	-1.45	46083	0.146
***On-narrow-focus*: *Mixed-tone***	***-0*.*42***	***-0*.*76 –-0*.*09***	***-2*.*49***	***46083***	***0*.*013***
***Post-narrow-focus*: *Mixed-tone***	***-0*.*5***	***-0*.*85 –-0*.*14***	***-2*.*72***	***46083***	***0*.*007***
**Group: Focus Condition: Tone Shape (Baseline: ASD: On-broad-focus: Level)**					
**ASD: Pre-narrow-focus: Rising**	0.15	-0.27–0.57	0.7	46083	0.483
**ASD: On-narrow-focus: Rising**	-0.1	-0.51–0.31	-0.46	46083	0.646
**ASD: Post-narrow-focus: Rising**	0	-0.43–0.44	0.01	46083	0.993
***ASD*: *Pre-narrow-focus*: *Low***	***0*.*86***	***0*.*34–1*.*38***	***3*.*23***	***46083***	***0*.*001***
**ASD: On-narrow-focus: Low**	0.07	-0.41–0.55	0.27	46083	0.785
**ASD: Post-focus: Low**	-0.24	-0.73–0.24	-0.98	46083	0.325
**Group: Focus Condition: Tone Shape (Baseline: TD: On-broad-focus: Level)**					
***TD*: *Pre-narrow-focus*: *Rising***	***0*.*59***	***0*.*16–1*.*02***	***2*.*69***	***46083***	***0*.*007***
**TD: On-narrow-focus: Rising**	0.09	-0.33–0.51	0.41	46083	0.68
**TD: Post-narrow-focus: Rising**	-0.04	-0.49–0.41	-0.17	46083	0.862
**TD: Pre-narrow-focus: Low**	0.15	-0.38–0.68	0.57	46083	0.57
**TD: On-narrow-focus: Low**	0.18	-0.31–0.67	0.7	46083	0.482
**TD: Post-narrow-focus: Low**	0.09	-0.40–0.59	0.37	46083	0.708

**Fig 4:**
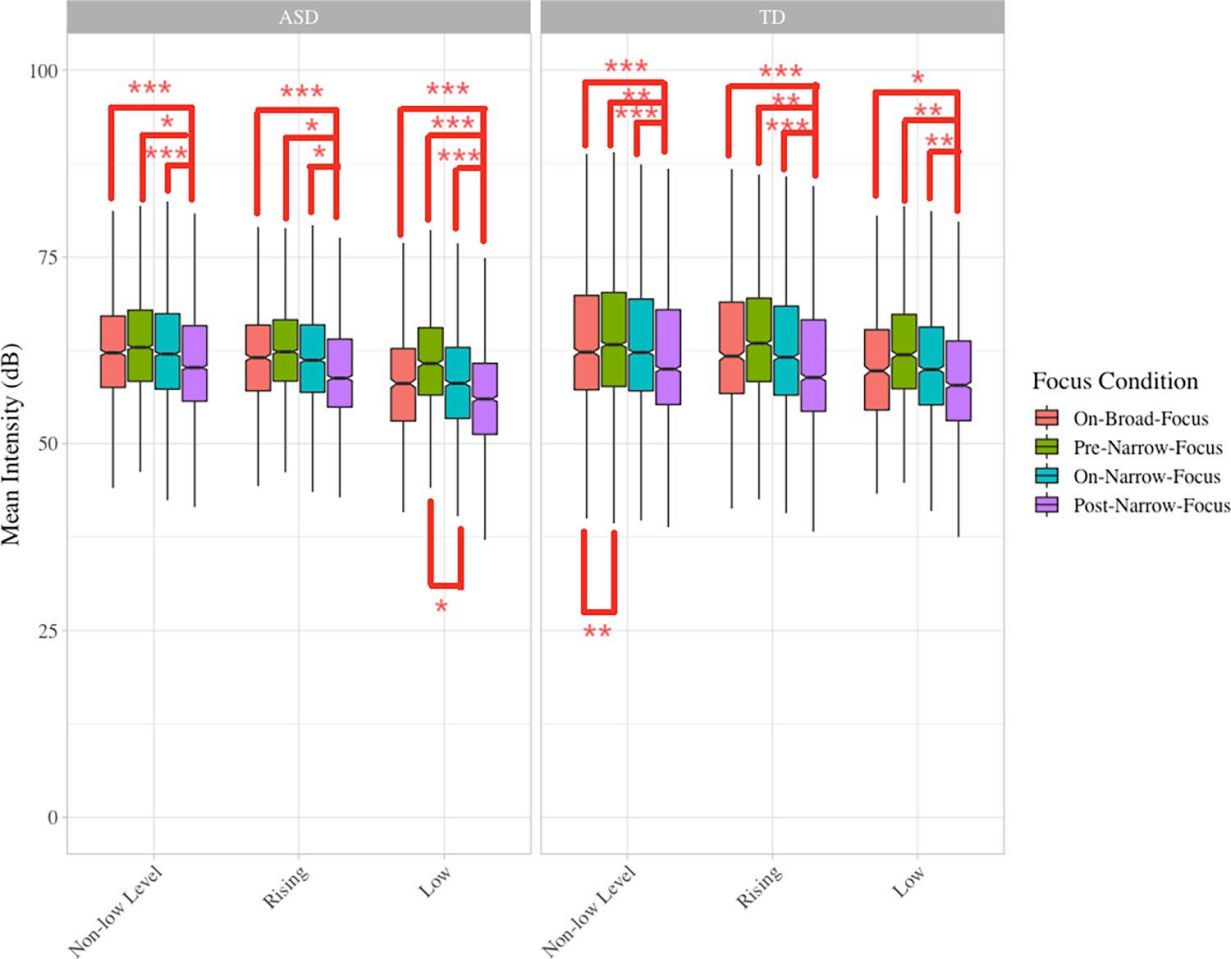
Boxplot of intensity. Note. Statistically significant differences between specific comparisons are indicated by asterisk: * indicates p < .05, ** indicates p < .01, and *** indicates p < .001.

Across groups and conditions, pre-narrow-focus syllables had the highest mean intensity and post-narrow-focus syllables had the lowest (Fig 4). Post-hoc comparisons showed no significant differences between the ASD and TD groups, but only significant differences between focus conditions within each group.

For level tones, in both groups, post-narrow-focus syllables had significantly lower intensity than pre-narrow-focus (ASD, *p* < 0.05; TD, *p* < 0.001), on-broad-focus and on-narrow-focus syllables (*p*s < 0.0001), and the difference between on-broad-focus and pre-narrow-focus syllables was also significant in the TD group (*p* < 0.001). For rising tones, similarly, post-narrow-focus syllables had significantly lower intensity than pre-narrow-focus (ASD, *p* < 0.05; TD, *p* < 0.001), on-broad-focus (*p*s < 0.001) and on-narrow-focus syllables (ASD, *p* < 0.05; TD, *p* = 0.0001). For syllables carrying low tones post-narrow-focus syllables had significantly lower intensity syllables in other focus conditions in the ASD (*p*s < 0.0001) and the TD group (*p*s < 0.05), but the on-narrow-focus syllables produced by the ASD group also had significantly lower intensity syllables than pre-narrow-focus syllables (*p* < 0.05).

## 4. Discussion

This study investigated the acoustic realization of focus by Cantonese-speaking autistic and TD children. Cantonese-speaking children with ASD employed the same acoustic cues to mark focus as their TD peers, but used them in different ways. Both the ASD and TD groups expanded *f*_0_ range and duration of the on-focus syllables while compressed the intensity of the post-focus syllables; nevertheless, the degree of on-focus expansion in the ASD group was smaller, and the two groups’ use of these acoustic cues show tone-specific patterns. Since the ASD and TD groups in the present study did not significantly differ from each other in IQ scores and language abilities, the clinical condition may be the primary factor that led to the results observed here.

In terms of *f*_0_ range, the autistic children in our study did not produce on-focus syllables with an expansion of *f*_0_ range compared to their TD peers. Autistic children did not only produce contour tones with significantly smaller *f*_0_ range than TD children at the post-narrow-focus position, but also low tones regardless of focus condition. In other conditions, the *f*_0_ range produced by the TD group was also slightly larger, though the difference did not reach statistical significance. At the first glance, this finding seems to be in line with early studies that reported prosodic production among the autistic population to be monotonic and machine-like (for review see [33]). However, since more recent studies suggest that the population with ASD tends to produce sing-songy prosody, we attribute these results to the autistic children’s failure to implement lexical and utterance prosody simultaneously, that is, to produce lexical tone accurately while marking information structure clearly. We will return to this point in the later discussion.

With regard to duration, while both the autistic and TD children produced long post-narrow-focus syllables, such lengthening may be due to the final lengthening (see [54] for instance). This is because two-thirds of the post-narrow-focus syllables fell on objects, namely, the last words of the sentences. It is worth noting as well that the post-narrow-focus syllables produced by TD were still shorter than syllables in the broad focus condition. In addition, children with ASD did not show evidently longer on-focus syllables compared to their TD peers. The present finding is more in line with the findings by Paul et al. and Grossman et al. that English speakers with ASD did not lengthen the stressed syllables enough. However, unlike in Diehl & Paul’s study, the autistic individuals in our study did not over-lengthen the syllables carrying no focus as pre-narrow-focus syllables produced by our autistic participants were the shortest. The differences between the present finding and Diehl & Paul’s study may be due to the differences in language background, namely, their participants were English speakers while ours were Cantonese speakers. Unlike English which used *f*_0_ patterns to mark utterance focus (cf. [55]), the major cue used for focus marking in Cantonese is the on-focus expansion of duration. Therefore, our participants with ASD still showed a tendency of on-focus lengthening, though not as sufficient as the TD peers.

In addition, we found an overall influence of lexical tones on the use of acoustic cues in both the ASD and TD groups, indicating that children face extra difficulties in marking prosodic focus in a tonal language. On the one hand, children need to vary *f*_0_ (and other acoustic cues) so as to produce accurate lexical tones. Previous studies have found that autistic children have speech-related deficits in tone production. Autistic children showed more *f*_0_ variations in imitating Mandarin lexical tones, but not in imitating non-speech stimuli [56]. On the other hand, they need to mark focus using acoustic cues involved in tone production. The difficulties in encoding both the lexical and focal function may have led to the smaller *f*_0_ range produced by the autistic children than the TD peers in general. The difficulties observed in focus marking especially for low tones in the present study may be due to the extra difficulty involved in low tone acquisition and production [57–60]. Moreover, for the ASD group, only on low tones were the on-narrow-focus syllables longer than on-broad-focus. Our results thus showed that the ASD group could mark focus using on focus expansion of duration only on the low tone. The low tone is reported to be among the shortest of Cantonese tones in its citation form, the lengthening in on-narrow-focus syllables may thus be more dramatic than other tones in focus marking due to its original short duration [61]. Also, it seems that final lengthening is more prominent on non-low level tones for both groups. It may be due to the fact that non-low level tones tend to have longer duration in the citation form and thus the final lengthening effect may be more prominent.

Based on these findings, we propose that Cantonese-speaking children with ASD did not use on-focus expansion in *f*_0_ range and intensity to mark focus, but showed some post-focus compression in these two cues. It is worth mentioning, however, unlike Mandarin and English, Cantonese is not a language with typical post-focus compression [42]. The seemingly smaller *f*_0_ range in post-focus syllables may alternatively be explained by the lack of *f*_0_ range expansion in the on-focus syllables, since in the ASD group no significance was found in *f*_0_ range between pre-focus and on-focus syllables when the embedded tones were level and rising tones and syllables on broad focus had the smallest *f*_0_ range when carrying low tones.

These findings allow us to answer our research questions by confirming that prosodic focus marking by Cantonese-speaking children with ASD is different from their TD peers. Furthermore, our results showed that the children with ASD indicate that they have problems encoding both the lexical and discourse information, leading to flattened lexical tones and insufficient on-focus expansion. Such deficits may be caused by differences found in the neural regions between the children with and without ASD. According to the neuro-imaging study conducted by Eigsti et al. [9], more generalized neural regions were activated in the ASD group compared to the TD group. Echoing Eigsti et al, Yu et al. [62] also found that different from the TD children, children with ASD did not show left-lateralized late negative response distinction when processing native lexical prosody. The reduced neural specialization involved in linguistic prosody processing may lead to the fact that the autistic population need cognitive control and resources in processing prosody, which is intrinsically challenging because it involves integration from multiple levels of language. As a result, the ASD group in the present study had some difficulties in marking focus and failed to keep as distinctive shapes of lexical tones as the TD peers while marking focus at the same time. ASD children were also reported to have difficulties in mapping acoustic cues and information structure [63]. Although they may use syntactic cues in comprehending focus, the ability to use prosodic cues to comprehend focus was significantly worse compared to their TD peers [64]. It has been reported that prosodic cues may help identify alternatives and affects implicature computation. The deficits in the mapping thus may lead to weaker identification of alternatives and implicature computation [65]. In turn, the deficit may lead to difficulties in using acoustic cues to mark information structure in speech production.

To conclude, this study has found that Cantonese-speaking children with ASD did not use as sufficient on-focus expansion to mark focus as their TD peers. The children with ASD also produced less distinctive *f*_0_ range for different tone shapes and focus conditions than TD children, but their focus-marking was not influenced by the prosodic complexity of the sentences. The findings of the present study have clinical implications. Our findings suggest that Cantonese-speaking children with ASD are not as sophisticated in prosodic focus marking as their TD peers, and therefore requires specific training, especially on how to retain distinctive *f*_0_ range for different tone shapes while marking focus more evidently.

## Supporting information

S1 File Stimulus list. (DOCX)

S1 Data Anonymous Data and the R script used for data analysis. (ZIP)
