# Supplementary material for: Focus-marking in a tonal language: Prosodic differences between Cantonese-speaking children with and without autism spectrum disorder
Source: PLoS One. 2024 Jul 19;19(7):e0306272. doi: 10.1371/journal.pone.0306272 (PMC11259269; doi:10.1371/journal.pone.0306272)
Supplement: S1 File — (DOCX) [file pone.0306272.s001.docx]

| Focus Condition | Precursor Sentence/Question | Target Sentence 1 |
| --- | --- | --- |
| Broad focus | /fat3 sɐŋ1 tsɔ2 mɛ1 si6 a3/  發生咗咩事啊？  What’s happening? | /tsœŋ1 saŋ1 tsa1 fei1 kei1/  [張生揸飛機]。  Mr. Cheung is flying the plane. |
| Narrow focus | /pin1 kɔ3 tsa1 fei1 kei1/  邊個揸飛機？  Who is flying the plane? | /tsœŋ1 saŋ1 tsa1 fei1 kei1/  [張生]揸飛機。  Mr. Cheung is flying the plane. |
|  | /tsœŋ1 saŋ1 mɐt1 jɛ5 fei1 kei1/  張生乜嘢飛機？  What is Mr. Cheung doing to the plane? | /tsœŋ1 saŋ1 tsa1 fei1 kei1/  張生[揸]飛機。  Mr. Cheung is flying the plane. |
|  | /tsœŋ1 saŋ1 tsa1 mɐt1 jɛ5/  張生揸乜嘢？  What is Mr. Cheung flying? | /tsœŋ1 saŋ1 tsa1 fei1 kei1/  張生揸[飛機]。  Mr. Cheung is flying the plane. |
| Contrastive focus | /tsʰɐn4 tʰai3 tsa1 fei1 kei1/  陳太揸飛機？  Is Mrs. Chan flying the plane? | /tsœŋ1 saŋ1 tsa1 fei1 kei1/  [張生]揸飛機。  Mr. Cheung is flying the plane. |
|  | /tsœŋ1 sɐŋ1 mai5 fei1 kei1/  張生買飛機？  Is Mr. Cheung buying the plane? | /tsœŋ1 saŋ1 tsa1 fei1 kei1/  張生[揸]飛機。  Mr. Cheung is flying the plane. |
|  | /tsœŋ1 sɐŋ1 tsa1 pa1 si2/  張生揸巴士？  Is Mr. Cheung driving a bus? | /tsœŋ1 saŋ1 tsa1 fei1 kei1/  張生揸[飛機]。  Mr. Cheung is flying the plane. |

| Focus Condition | Precursor Sentence/Question | Target Sentence 2 |
| --- | --- | --- |
| Broad focus | /fat3 sɐŋ1 tsɔ2 mɛ1 si6 a3/  發生咗咩事啊？  What’s happening? | /jyn2 jyn2 mɔ2 kɐu2 tsɐi2/  [婉婉摸狗仔]。  Yuen-Yuen is petting the dog. |
| Narrow focus | /pin1 kɔ3 mɔ2 kɐu2 tsɐi2/  邊個摸狗仔？  Who is petting the dog? | /jyn2 jyn2 mɔ2 jyn2 jyn2 /  [婉婉]摸狗仔。  Yuen-Yuen is petting the dog. |
|  | /jyn2 jyn2 mɐt1 jɛ5 kɐu2 tsɐi2/  婉婉乜嘢狗仔？  What is Yuen-Yuen doing to the dog? | /jyn2 jyn2 mɔ2 kɐu2 tsɐi2/  婉婉[摸]狗仔。  Yuen-Yuen is petting the dog. |
|  | /jyn2 jyn2 mɔ2 mɐt1 jɛ5/  婉婉摸乜嘢?  What is Yuen-Yuen petting? | /jyn2 jyn2 mɔ2 kɐu2 tsɐi2/  婉婉摸[狗仔]。  Yuen-Yuen is petting the dog. |
| Contrastive focus | /kɔ4 kɔ4 mɔ2 kɐu2 tsɐi2/  哥哥摸狗仔？  Is brother petting the dog? | /jyn2 jyn2 mɔ2 kɐu2 tsɐi2/  [婉婉]摸狗仔。  Yuen-Yuen is petting the dog. |
|  | /jyn2 jyn2 nau6 kɐu2 tsɐi2/  婉婉鬧狗仔？  Is Yuen-Yuen yelling at the dog? | /jyn2 jyn2 mɔ2 kɐu2 tsɐi2/  婉婉[摸]狗仔。  Yuen-Yuen is petting the dog. |
|  | /jyn2 jyn2 mɔ2 tai6 tsœŋ6/  婉婉摸大象？  Is Yuen-Yuen petting the elephant? | /jyn2 jyn2 mɔ2 kɐu2 tsɐi2/  婉婉摸[狗仔]。  Yuen-Yuen is petting the dog. |

| Focus Condition | Precursor Sentence/Question | Target Sentence 3 |
| --- | --- | --- |
| Broad focus | /fat3 sɐŋ1 tsɔ2 mɛ1 si6 a3/  發生咗咩事啊？  What’s happening? | /sɐu3 sɐu3 sɛk3 pak3 pak3/  [秀秀錫伯伯]。  Sau-Sau is kissing her uncle. |
| Narrow focus | /pin1 kɔ3 sɛk3 pak3 pak3/  邊個錫伯伯？  Who is kissing her uncle? | /sɐu3 sɐu3 sɛk3 pak3 pak3/  [秀秀]錫伯伯。  Sau-Sau is kissing her uncle. |
|  | /sɐu3 sɐu3 mɐt1 jɛ5 pak3 pak3/  秀秀乜嘢伯伯？  What is Sau-Sau doing to her uncle? | /sɐu3 sɐu3 sɛk3 pak3 pak3/  秀秀[錫]伯伯。  Sau-Sau is kissing her uncle. |
|  | /sɐu3 sɐu3 sɛk3 pin1 kɔ3/  秀秀錫邊個?  Who is Sau-Sau kissing? | /sɐu3 sɐu3 sɛk3 pak3 pak3/  秀秀錫[伯伯]。  Sau-Sau is kissing her uncle. |
| Contrastive focus | /kɔ4 kɔ4 sɛk3 pak3 pak3/  哥哥錫伯伯？  Is my brother kissing her uncle? | /sɐu3 sɐu3 sɛk3 pak3 pak3/  [秀秀]錫伯伯。  Sau-Sau is kissing her uncle. |
|  | /sɐu3 sɐu3 kiu3 pak3 pak3/  秀秀叫伯伯？  Is Sau-Sau calling her uncle? | /sɐu3 sɐu3 sɛk3 pak3 pak3/  秀秀[錫]伯伯。  Sau-Sau is kissing her uncle. |
|  | /sɐu3 sɐu3 sɛk8 pʰɔ4 pʰɔ4/  秀秀錫婆婆？  Is Sau-Sau kissing the old lady? | /sɐu3 sɐu3 sɛk3 pak3 pak3/  秀秀錫[伯伯]。  Sau-Sau is kissing her uncle. |

| Focus Condition | Precursor Sentence/Question | Target Sentence 4 |
| --- | --- | --- |
| Broad focus | /fat3 sɐŋ1 tsɔ2 mɛ1 si6 a3/  發生咗咩事啊？  What’s happening? | /ma4 ma4 fu4 maŋ4 jɐn4/  [嫲嫲扶盲人] 。  Grandma is helping the blind. |
| Narrow focus | /pin1 kɔ3 fu4 maŋ4 jɐn4/  邊個扶盲人？  Who is helping the blind? | /ma4 ma4 fu4 maŋ4 jɐn4/  [嫲嫲]扶盲人 。  Grandma is helping the blind. |
|  | /ma4 ma4 mɐt1 jɛ5 maŋ4 jɐn4/  嫲嫲乜嘢盲人？  What is grandma doing to the blind? | /ma4 ma4 fu4 maŋ4 jɐn4/  嫲嫲[扶]盲人 。  Grandma is helping the blind. |
|  | /ma4 ma4 fu4 pin1 kɔ3/  嫲嫲扶邊個？  Who is grandma helping? | /ma4 ma4 fu4 maŋ4 jɐn4/  嫲嫲扶[盲人] 。  Grandma is helping the blind. |
| Contrastive focus | /pak3 pak3 fu4 maŋ4 jɐn4/  伯伯扶盲人？  Is uncle helping the blind? | /ma4 ma4 fu4 maŋ4 jɐn4/  [嫲嫲]扶盲人 。  Grandma is helping the blind. |
|  | /ma4 ma4 tʰɵy1 maŋ4 jɐn4/  嫲嫲推盲人？  Is grandma pushing the blind? | /ma4 ma4 fu4 maŋ4 jɐn4/  嫲嫲[扶]盲人 。  Grandma is helping the blind. |
|  | /ma4 ma4 fu4 jɛ4 jɛ4/  嫲嫲扶爺爺？  Is grandma helping grandpa? | /ma4 ma4 fu4 maŋ4 jɐn4/  嫲嫲扶[盲人] 。  Grandma is helping the blind. |

| Focus Condition | Precursor Sentence/Question | Target Sentence 5 |
| --- | --- | --- |
| Broad focus | /fat3 sɐŋ1 tsɔ2 mɛ1 si6 a3/  發生咗咩事啊？  What’s happening? | /ŋa5 ŋa5 mai5 pʰou5 pʰou5/  [雅雅買泡泡]。  Nga-Nga is buying the bubble. |
| Narrow focus | /pin1 kɔ3 mai5 pʰou5 pʰou5/  邊個買泡泡？  Who is buying the bubble? | /ŋa5 ŋa5 mai5 pʰou5 pʰou5/  [雅雅]買泡泡。  Nga-Nga is buying the bubble. |
|  | /ŋa5 ŋa5 mɐt1 jɛ5 pʰou5 pʰou5/  雅雅乜嘢泡泡？  What is Nga-Nga doing to the bubble? | /ŋa5 ŋa5 mai5 pʰou5 pʰou5/  雅雅[買]泡泡。  Nga-Nga is buying the bubble. |
|  | /ŋa5 ŋa5 mai5 mɐt1 jɛ5/  雅雅買乜嘢？  What is Nga-Nga buying? | /ŋa5 ŋa5 mai5 pʰou5 pʰou5/  雅雅買[泡泡]。  Nga-Nga is buying the bubble. |
| Contrastive focus | /jɛ4 jɛ4 mai5 pʰou5 pʰou5/  爺爺買泡泡？  Is grandpa buying the bubble? | /ŋa5 ŋa5 mai5 pʰou5 pʰou5/  [雅雅]買泡泡。  Nga-Nga is buying the bubble. |
|  | /ŋa5 ŋa5 tsʰɵy1 pʰou5 pʰou5/  雅雅吹泡泡？  Is Nga-Nga blowing the bubble? | /ŋa5 ŋa5 mai5 pʰou5 pʰou5/  雅雅[買]泡泡。  Nga-Nga is buying the bubble. |
|  | /ŋa5 ŋa5 mai5 pʰɪŋ4 kwɔ2/  雅雅買蘋果？  Is Nga-Nga buying the apple? | /ŋa5 ŋa5 mai5 pʰou5 pʰou5/  雅雅買[泡泡]。  Nga-Nga is buying the bubble. |

| Focus Condition | Precursor Sentence/Question | Target Sentence 6 |
| --- | --- | --- |
| Broad focus | /fat3 sɐŋ1 tsɔ2 mɛ1 si6 a3/  發生咗咩事啊？  What’s happening? | /lɔk6 lɔk6 wak6 jyt6 lœŋ6/  [樂樂畫月亮]。  Lok-Lok is drawing the moon. |
| Narrow focus | /pin1 kɔ3 wak6 jyt6 lœŋ6/  邊個畫月亮？  Who is drawing the moon? | /lɔk6 lɔk6 wak6 jyt6 lœŋ6/  [樂樂]畫月亮。  Lok-Lok is drawing the moon. |
|  | /lɔk6 lɔk6 mɐt1 jɛ5 jyt6 lœŋ6/  樂樂乜嘢月亮？  What is Lok-Lok doing to the moon? | /lɔk6 lɔk6 wak6 jyt6 lœŋ6/  樂樂[畫]月亮。  Lok-Lok is drawing the moon. |
|  | /lɔk6 lɔk6 wak6 mɐt1 jɛ5/  樂樂畫乜嘢？  What is Lok-Lok drawing? | /lɔk6 lɔk6 wak6 jyt6 lœŋ6/  樂樂畫[月亮]。  Lok-Lok is drawing the moon. |
| Contrastive focus | /ma2 ma2 wak6 jyt6 lœŋ6/  媽媽畫月亮？  Is mum drawing the moon? | /lɔk6 lɔk6 wak6 jyt6 lœŋ6/  [樂樂]畫月亮。  Lok-Lok is drawing the moon. |
|  | /lɔk6 lɔk6 tʰɐi2 jyt6 lœŋ6/  樂樂睇月亮？  Is Lok-Lok looking at the moon? | /lɔk6 lɔk6 wak6 jyt6 lœŋ6/  樂樂[畫]月亮。  Lok-Lok is drawing the moon. |
|  | /lɔk6 lɔk6 wak6 tʰai3 jœŋ4/  樂樂畫太陽？  Is Lok-Lok drawing the sun? | /lɔk6 lɔk6 wak6 jyt6 lœŋ6/  樂樂畫[月亮]。  Lok-Lok is drawing the moon. |

| Focus Condition | Precursor Sentence/Question | Target Sentence 7 |
| --- | --- | --- |
| Broad focus | /fat3 sɐŋ1 tsɔ2 mɛ1 si6 a3/  發生咗咩事啊？  What’s happening? | /sʊk1 sʊk1 sɐi2 sɵy2 kwɔ2/  [叔叔洗水果] 。  Uncle is washing the fruit. |
| Narrow focus | /pin1 kɔ3 sɐi2 sɵy2 kwɔ2/  邊個洗水果？  Who is washing the fruit? | /sʊk1 sʊk1 sɐi2 sɵy2 kwɔ2/  [叔叔]洗水果 。  Uncle is washing the fruit. |
|  | /sʊk1 sʊk1 mɐt1 jɛ5 sɵy2 kwɔ2/  叔叔乜嘢水果？  What is uncle doing to the fruit? | /sʊk1 sʊk1 sɐi2 sɵy2 kwɔ2/  叔叔[洗]水果 。  Uncle is washing the fruit. |
|  | /sʊk1 sʊk1 sɐi2 mɐt1 jɛ5/  叔叔洗乜嘢？  What is uncle washing? | /sʊk1 sʊk1 sɐi2 sɵy2 kwɔ2/  叔叔洗[水果] 。  Uncle is washing the fruit. |
| Contrastive focus | /sɐm2 sɐm2 sɐi2 sɵy2 kwɔ2/  嬸嬸洗水果？  Is aunt washing the fruit? | /sʊk1 sʊk1 sɐi2 sɵy2 kwɔ2/  [叔叔]洗水果 。  Uncle is washing the fruit. |
|  | /sʊk1 sʊk1 mai6 sɵy2 kwɔ2/  叔叔賣水果？  Is uncle selling the fruit? | /sʊk1 sʊk1 sɐi2 sɵy2 kwɔ2/  叔叔[洗]水果 。  Uncle is washing the fruit. |
|  | /sʊk1 sʊk1 sɐi2 sɔ1 tsʰɔi2/  叔叔洗蔬菜？  Is uncle washing the vegetable? | /sʊk1 sʊk1 sɐi2 sɵy2 kwɔ2/  叔叔洗[水果] 。  Uncle is washing the fruit. |

| Focus Condition | Precursor Sentence/Question | Target Sentence 8 |
| --- | --- | --- |
| Broad focus | /fat3 sɐŋ1 tsɔ2 mɛ1 si6 a3/  發生咗咩事啊？  What’s happening? | /piu2 tsɛ2 tsɐp1 sy1 pau1/  [表姐執書包] 。  Cousin is packing the schoolbag. |
| Narrow focus | /pin1 kɔ3 tsɐp1 sy1 pau1/  邊個執書包？  Who is packing the schoolbag? | /piu2 tsɛ2 tsɐp1 sy1 pau1/  [表姐]執書包 。  Cousin is packing the schoolbag. |
|  | /piu2 tsɛ2 mɐt1 jɛ5 sy1 pau1/  表姐乜嘢書包？  What is cousin doing to the schoolbag? | /piu2 tsɛ2 tsɐp1 sy1 pau1/  表姐[執]書包 。  Cousin is packing the schoolbag. |
|  | /piu2 tsɛ2 tsɐp1 mɐt1 jɛ5/  表姐執乜嘢？  What is cousin packing? | /piu2 tsɛ2 tsɐp1 sy1 pau1/  表姐執[書包] 。  Cousin is packing the schoolbag. |
| Contrastive focus | /tɐi6 tɐi6 tsɐp1 sy1 pau1/  弟弟執書包？  Is brother packing the schoolbag? | /piu2 tsɛ2 tsɐp1 sy1 pau1/  [表姐]執書包 。  Cousin is packing the schoolbag. |
|  | /piu2 tsɛ2 sɐi2 sy1 pau1/  表姐洗書包？  Is cousin washing the schoolbag? | /piu2 tsɛ2 tsɐp1 sy1 pau1/  表姐[執]書包 。  Cousin is packing the schoolbag. |
|  | /piu2 tsɛ2 tsɐp1 ji1 kwɐi6/  表姐執衣櫃？  Is cousin packing the wardrobe? | /piu2 tsɛ2 tsɐp1 sy1 pau1/  表姐執[書包] 。  Cousin is packing the schoolbag. |

| Focus Condition | Precursor Sentence/Question | Target Sentence 9 |
| --- | --- | --- |
| Broad focus | /fat3 sɐŋ1 tsɔ2 mɛ1 si6 a3/  發生咗咩事啊？  What’s happening? | /pui3 pui3 tsʰɵy1 fʊŋ1 tsʰɛ1/  [貝貝吹風車] 。  Pui-Pui is blowing the pinwheel. |
| Narrow focus | /pin1 kɔ3 tsʰɵy1 fʊŋ1 tsʰɛ1/  邊個吹風車？  Who is blowing the pinwheel? | /pui3 pui3 tsʰɵy1 fʊŋ1 tsʰɛ1/  [貝貝]吹風車 。  Pui-Pui is blowing the pinwheel. |
|  | /pui3 pui3 mɐt1 jɛ5 fʊŋ1 tsʰɛ1/  貝貝乜嘢風車？  What is Pui-Pui doing to the pinwheel? | /pui3 pui3 tsʰɵy1 fʊŋ1 tsʰɛ1/  貝貝[吹]風車 。  Pui-Pui is blowing the pinwheel. |
|  | /pui3 pui3 tsʰɵy1 mɐt1 jɛ5/  貝貝吹乜嘢？  What is Pui-Pui blowing? | /pui3 pui3 tsʰɵy1 fʊŋ1 tsʰɛ1/  貝貝吹[風車] 。  Pui-Pui is blowing the pinwheel. |
| Contrastive focus | /mɐn4 mɐn4 tsʰɵy1 fʊŋ1 tsʰɛ1/  文文吹風車？  Is Man-Man blowing the pinwheel? | /pui3 pui3 tsʰɵy1 fʊŋ1 tsʰɛ1/  [貝貝]吹風車 。  Pui-Pui is blowing the pinwheel. |
|  | /pui3 pui3 tsɐp1 fʊŋ1 tsʰɛ1 /  貝貝執風車？  Is Man-Man picking up the pinwheel? | /pui3 pui3 tsʰɵy1 fʊŋ1 tsʰɛ1/  貝貝[吹]風車 。  Pui-Pui is blowing the pinwheel. |
|  | /pui3 pui3 tsʰɵy1 pʰou5 pʰou5/  貝貝吹泡泡？  Is Man-Man blowing the bubbles? | /pui3 pui3 tsʰɵy1 fʊŋ1 tsʰɛ1/  貝貝吹[風車] 。  Pui-Pui is blowing the pinwheel. |

| Focus Condition | Precursor Sentence/Question | Target Sentence 10 |
| --- | --- | --- |
| Broad focus | /fat3 sɐŋ1 tsɔ2 mɛ1 si6 a3/  發生咗咩事啊？  What’s happening? | /pak3 pak3 tsɪŋ2 kau2 tsi2/  [伯伯整餃子]。  Uncle is making the dumplings. |
| Narrow focus | /pin1 kɔ3 tsɪŋ2 kau2 tsi2/  邊個整餃子？  Who is making the dumplings? | /pak3 pak3 tsɪŋ2 kau2 tsi2/  [伯伯]整餃子。  Uncle is making the dumplings. |
|  | /pak3 pak3 mɐt1 jɛ5 kau2 tsi2/  伯伯乜嘢餃子？  What is uncle doing to the dumplings? | /pak3 pak3 tsɪŋ2 kau2 tsi2/  伯伯[整]餃子。  Uncle is making the dumplings. |
|  | /pak3 pak3 tsɪŋ2 mɐt1 jɛ5/  伯伯整乜嘢？  What is uncle making? | /pak3 pak3 tsɪŋ2 kau2 tsi2/  伯伯整[餃子]。  Uncle is making the dumplings. |
| Contrastive focus | /ji4 ji4 tsɪŋ2 kau2 tsi2/  姨姨整餃子？  Is aunt making the dumplings? | /pak3 pak3 tsɪŋ2 kau2 tsi2/  [伯伯]整餃子。  Uncle is making the dumplings. |
|  | /pak3 pak3 sik6 kau2 tsi2/  伯伯食餃子？  Is uncle having the dumplings？ | /pak3 pak3 tsɪŋ2 kau2 tsi2/  伯伯[整]餃子。  Uncle is making the dumplings. |
|  | /pak3 pak3 tsɪŋ2 tʰɔŋ1 jyn4/  伯伯整湯圓？  Is uncle making sweet dumplings? | /pak3 pak3 tsɪŋ2 kau2 tsi2/  伯伯整[餃子]。  Uncle is making the dumplings. |

| Focus Condition | Precursor Sentence/Question | Target Sentence 11 |
| --- | --- | --- |
| Broad focus | /fat3 sɐŋ1 tsɔ2 mɛ1 si6 a3/  發生咗咩事啊？  What’s happening? | /jɐn1 jɐn1 wɐi3 tsʰam3 tsʰam3/  [欣欣餵杉杉]。  Yan-Yan is feeding Cham-Cham. |
| Narrow focus | /pin1 kɔ3 wɐi3 tsʰam3 tsʰam3/  邊個餵杉杉？  Who is feeding Cham-Cham? | /jɐn1 jɐn1 wɐi3 tsʰam3 tsʰam3/  [欣欣]餵杉杉。  Yan-Yan is feeding Cham-Cham. |
|  | /jɐn1 jɐn1 mɐt1 jɛ5 tsʰam3 tsʰam3/  欣欣乜嘢杉杉？  What is Yan-Yan doing to Cham Cham? | /jɐn1 jɐn1 wɐi3 tsʰam3 tsʰam3/  欣欣[餵]杉杉。  Yan-Yan is feeding Cham-Cham. |
|  | /jɐn1 jɐn1 wɐi3 pin1 kɔ3/  欣欣餵邊個？  Who is Yan-Yan feeding? | /jɐn1 jɐn1 wɐi3 tsʰam3 tsʰam3/  欣欣餵[杉杉]。  Yan-Yan is feeding Cham-Cham. |
| Contrastive focus | /fei1 fei1 wɐi3 tsʰam3 tsʰam3/  菲菲餵杉杉？  Is Fei-Fei feeding Cham-Cham? | /jɐn1 jɐn1 wɐi3 tsʰam3 tsʰam3/  [欣欣]餵杉杉。  Yan-Yan is feeding Cham-Cham. |
|  | /jɐn1 jɐn1 pʰou5 tsʰam3 tsʰam3/  欣欣抱杉杉？  Is Yan-Yan hugging Cham-Cham? | /jɐn1 jɐn1 wɐi3 tsʰam3 tsʰam3/  欣欣[餵]杉杉。  Yan-Yan is feeding Cham-Cham. |
|  | /jɐn1 jɐn1 wɐi3 tin1 tin1/  欣欣餵天天？  Is Yan-Yan feeding Tin-Tin? | /jɐn1 jɐn1 wɐi3 tsʰam3 tsʰam3/  欣欣餵[杉杉]。  Yan-Yan is feeding Cham-Cham. |

| Focus Condition | Precursor Sentence/Question | Target Sentence 12 |
| --- | --- | --- |
| Broad focus | /fat3 sɐŋ1 tsɔ2 mɛ1 si6 a3/  發生咗咩事啊？  What’s happening? | /piu2 mui2 tsan3 jin3 jin3/  [表妹讚燕燕]。  Cousin is praising Yin-Yin. |
| Narrow focus | /pin1 kɔ3 tsan3 jin3 jin3/  邊個讚燕燕？  Who is praising Yin-Yin? | /piu2 mui2 tsan3 jin3 jin3/  [表妹]讚燕燕。  Cousin is praising Yin-Yin. |
|  | /piu2 mui2 mɐt1 jɛ5 jin3 jin3/  表妹乜嘢燕燕？  What is cousin doing to Yin-Yin? | /piu2 mui2 tsan3 jin3 jin3/  表妹[讚]燕燕。  Cousin is praising Yin-Yin. |
|  | /piu2 mui2 tsan3 pin1 kɔ3/  表妹讚邊個？  Who is cousin praising? | /piu2 mui2 tsan3 jin3 jin3/  表妹讚[燕燕]。  Cousin is praising Yin-Yin. |
| Contrastive focus | /ka1 tsɛ2 tsan3 jin3 jin3/  家姐讚燕燕？  Is sister praising Yin-Yin? | /piu2 mui2 tsan3 jin3 jin3/  [表妹]讚燕燕。  Cousin is praising Yin-Yin. |
|  | /piu2 mui2 nau6 jin3 jin3/  表妹鬧燕燕？  Is cousin yelling at Yin-Yin? | /piu2 mui2 tsan3 jin3 jin3/  表妹[讚]燕燕。  Cousin is praising Yin-Yin. |
|  | /piu2 mui2 tsan3 hou6 hou6/  表妹讚浩浩？  Is cousin praising Ho-Ho? | /piu2 mui2 tsan3 jin3 jin3/  表妹讚[燕燕]。  Cousin is praising Yin-Yin. |

| Focus Condition | Precursor Sentence/Question | Target Sentence 13 |
| --- | --- | --- |
| Broad focus | /fat3 sɐŋ1 tsɔ2 mɛ1 si6 a3/  發生咗咩事啊？  What’s happening? | /wɐi5 wɐi5 wat3 pui3 hɔk3/  [偉偉挖貝殼]。  Wai-Wai is digging the seashell. |
| Narrow focus | /pin1 kɔ3 wat3 pui3 hɔk3/  邊個挖貝殼？  Who is digging the seashell? | /wɐi5 wɐi5 wat3 pui3 hɔk3/  [偉偉]挖貝殼。  Wai-Wai is digging the seashell. |
|  | /wɐi5 wɐi5 mɐt1 jɛ5 pui3 hɔk3/  偉偉乜嘢貝殼？  What is Wai-Wai doing to the seashell? | /wɐi5 wɐi5 wat3 pui3 hɔk3/  偉偉[挖]貝殼。  Wai-Wai is digging the seashell. |
|  | /wɐi5 wɐi5 wat3 mɐt1 jɛ5/  偉偉挖乜嘢？  What is Wai-Wai digging? | /wɐi5 wɐi5 wat3 pui3 hɔk3/  偉偉挖[貝殼]。  Wai-Wai is digging the seashell. |
| Contrastive focus | /lɐm4 lɐm4 wat3 pui3 hɔk3/  琳琳挖貝殼？  Is Lam-Lam digging the seashell? | /wɐi5 wɐi5 wat3 pui3 hɔk3/  [偉偉]挖貝殼。  Wai-Wai is digging the seashell. |
|  | /wɐi5 wɐi5 tsɐp1 pui3 hɔk3/  偉偉執貝殼？  Is Wai-Wai picking up the seashell? | /wɐi5 wɐi5 wat3 pui3 hɔk3/  偉偉[挖]貝殼。  Wai-Wai is digging the seashell. |
|  | /wɐi5 wɐi5 wat3 pʰɔŋ4 hai5/  偉偉挖螃蟹？  Is Wai-Wai digging the crab? | /wɐi5 wɐi5 wat3 pui3 hɔk3/  偉偉挖[貝殼]。  Wai-Wai is digging the seashell. |

| Focus Condition | Precursor Sentence/Question | Target Sentence 14 |
| --- | --- | --- |
| Broad focus | /fat3 sɐŋ1 tsɔ2 mɛ1 si6 a3/  發生咗咩事啊？  What’s happening? | /siu2 kwɔŋ2 tsʰyn4 lam4 kʰɐu4/  [小廣傳籃球]。  Siu-Kwong is throwing the basketball. |
| Narrow focus | /pin1 kɔ3 tsʰyn4 lam4 kʰɐu4/  邊個傳籃球？  Who is throwing the basketball? | /siu2 kwɔŋ2 tsʰyn4 lam4 kʰɐu4/  [小廣]傳籃球。  Siu-Kwong is throwing the basketball. |
|  | /siu2 kwɔŋ2 mɐt1 jɛ5 lam4 kʰɐu4/  小廣乜嘢籃球？  What is Siu-Kwong doing to the basketball? | /siu2 kwɔŋ2 tsʰyn4 lam4 kʰɐu4/  小廣[傳]籃球。  Siu-Kwong is throwing the basketball. |
|  | /siu2 kwɔŋ2 tsʰyn4 mɐt1 jɛ5/  小廣傳乜嘢？  What is Siu-Kwong throwing? | /siu2 kwɔŋ2 tsʰyn4 lam4 kʰɐu4/  小廣傳[籃球]。  Siu-Kwong is throwing the basketball. |
| Contrastive focus | /tsɛ2 tsɛ2 tsʰyn4 lam4 kʰɐu4/  姐姐傳籃球？  Is sister throwing the basketball? | /siu2 kwɔŋ2 tsʰyn4 lam4 kʰɐu4/  [小廣]傳籃球。  Siu-Kwong is throwing the basketball. |
|  | /siu2 kwɔŋ2 tsɐp1 lam4 kʰɐu4/  小廣執籃球？  Is Siu-Kwong picking up the basketball? | /siu2 kwɔŋ2 tsʰyn4 lam4 kʰɐu4/  小廣[傳]籃球。  Siu-Kwong is throwing the basketball. |
|  | /siu2 kwɔŋ2 tsʰyn4 tsʊk1 kʰɐu4/  小廣傳足球？  Is Siu-Kwong throwing the football? | /siu2 kwɔŋ2 tsʰyn4 lam4 kʰɐu4/  小廣傳[籃球]。  Siu-Kwong is throwing the basketball. |

| Focus Condition | Precursor Sentence/Question | Target Sentence 15 |
| --- | --- | --- |
| Broad focus | /fat3 sɐŋ1 tsɔ2 mɛ1 si6 a3/  發生咗咩事啊？  What’s happening? | /tsʰɵy3 tsʰɵy3 tsʰa4 ŋɐu4 jɐu4/  [翠翠搽牛油]。  Tsui-Tsui is spreading the butter. |
| Narrow focus | /pin1 kɔ3 tsʰa4 ŋɐu4 jɐu4/  邊個搽牛油？  Who is spreading the butter? | /tsʰɵy3 tsʰɵy3 tsʰa4 ŋɐu4 jɐu4/  [翠翠]搽牛油。  Tsui-Tsui is spreading the butter. |
|  | /tsʰɵy3 tsʰɵy3 mɐt1 jɛ5 ŋɐu4 jɐu4/  翠翠乜嘢牛油？  What is Tsui-Tsui doing to the butter? | /tsʰɵy3 tsʰɵy3 tsʰa4 ŋɐu4 jɐu4/  翠翠[搽]牛油。  Tsui-Tsui is spreading the butter. |
|  | /tsʰɵy3 tsʰɵy3 tsʰa4 mɐt1 jɛ5/  翠翠搽乜嘢？  What is Tsui-Tsui spreading? | /tsʰɵy3 tsʰɵy3 tsʰa4 ŋɐu4 jɐu4/  翠翠搽[牛油]。  Tsui-Tsui is spreading the butter. |
| Contrastive focus | /wa4 wa4 tsʰa4 ŋɐu4 jɐu4/  華華搽牛油？  Is Wah-Wah spreading the butter? | /tsʰɵy3 tsʰɵy3 tsʰa4 ŋɐu4 jɐu4/  [翠翠]搽牛油。  Tsui-Tsui is spreading the butter. |
|  | /tsʰɵy3 tsʰɵy3 sik6 ŋɐu4 jɐu4/  翠翠食牛油？  Is Tsui-Tsui eating the butter? | /tsʰɵy3 tsʰɵy3 tsʰa4 ŋɐu4 jɐu4/  翠翠[搽]牛油。  Tsui-Tsui is spreading the butter. |
|  | /tsʰɵy3 tsʰɵy3 tsʰa4 hœŋ1 sɵy2/  翠翠搽香水？  Is Tsui-Tsui spreading the perfume? | /tsʰɵy3 tsʰɵy3 tsʰa4 ŋɐu4 jɐu4/  翠翠搽[牛油]。  Tsui-Tsui is spreading the butter. |
